# MQTT_UAD: MQTT Under Attack Dataset. A public dataset for the detection of attacks in IoT networks using MQTT protocol

**DOI:** 10.1016/j.dib.2025.112167

**Published:** 2025-10-10

**Authors:** Jose Aveleira-Mata, Héctor Alaiz-Moretón, Martín Bayón-Guitérrez, María Teresa Gacía-Ordás, Natalia Prieto-Fernandez, Isaías García-Rodríguez

**Affiliations:** University of León, Department of Electric, Systems and Automatics Engineering, León, Spain

**Keywords:** IoT security, MQTT protocol, Intrusion detection system, Cyberattack dataset, Anomaly detection

## Abstract

Internet of Things (IoT) systems have become increasingly popular in recent years, leading to a growing need to ensure their security. The diversity of devices and protocols, combined with the limited computational power of many of these devices, makes them especially vulnerable to a wide range of threats. Detecting cyberattacks is therefore essential to ensure the safe operation of systems that IoT devices.

In this work, we present MQTT UAD (MQTT Under Attack Dataset), a dataset specifically designed to analyze security in networks based on the MQTT protocol. This dataset has already been used to train intrusion detection models, but it had not been published independently or described in technical detail. Due to the interest it has generated, we provide a clear name and a full description of how it was created, processed, and structured.

To build the dataset, we set up a realistic test environment where different attacks against the MQTT protocol were simulated, including Denial of Service (DoS), Man-in-the-Middle (MitM), and Intrusion attacks. Network traffic was captured during these tests, and the malicious frames were tagged. Finally, we validate the MQTT UAD for developing machine learning models aimed at detecting attacks in MQTT networks.

## Graphical abstract

**Figure:**
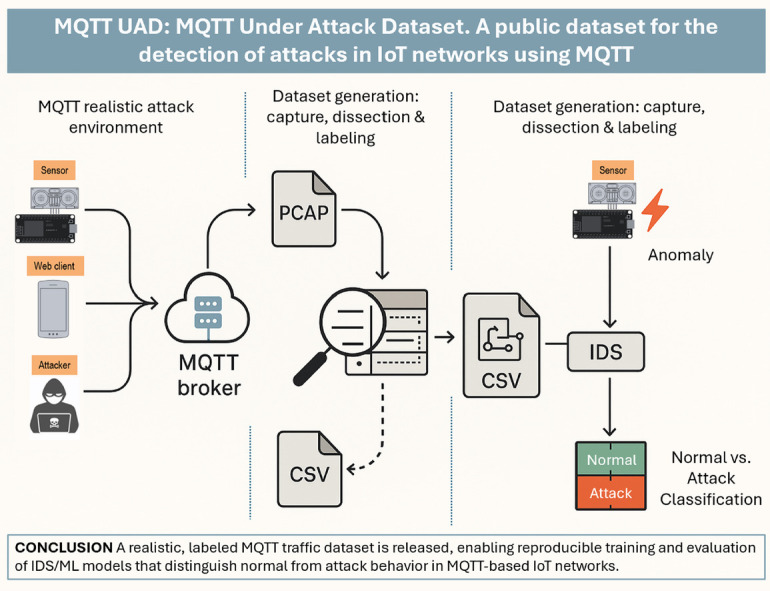

Specifications Table

**Table:** 

Subject	Computer Sciences
Specific subject area	IoT security with MQTT protocol: labelled network traffic for DoS, Man-in-the-Middle, and Intrusion attack detection in real MQTT testbeds.
Type of data	Three tables in CSV format Data format: Processed/labelled (CSV, 67 features per frame)
Data collection	The MQTT_UAD dataset is designed to evaluate security in networks using the MQTT protocol. It includes three files with normal and malicious traffic, focusing on key vulnerabilities such as Denial of Service, Man-in-the-Middle, and Intrusion attacks. The dataset is intended for developing and testing Machine Learning-based Intrusion Detection Systems in IoT environments—both domestic and industrial—and provides curated, labelled tables to support reproducible training, validation, and benchmarking.
Data source location	Country: Spain. City/Region: León. Institution: University of León (RIASC / SECOMUCI). Real IoT laboratory testbed.
Data accessibility	Repository name: Figshare. Data identification number: 10.6084/m9.figshare.24420958 Direct URL to data: https://figshare.com/articles/dataset/MQTT_UAD_MQTT_Under_Attack_Dataset_A_public_dataset_for_the_detection_of_attacks_in_IoT_networks_using_MQTT_protocol/24420958
Related research article	Alaiz-Moreton, Hector, Aveleira-Mata, Jose, Ondicol-Garcia, Jorge, Muñoz-Castañeda, Angel Luis, García, Isaías, Benavides, Carmen, Multiclass Classification Procedure for Detecting Attacks on MQTT-IoT Protocol, Complexity, 2019, 6516,253, 11 pages, 2019. https://doi.org/10.1155/2019/6516253

## 1 Value of the Data

• Protocol-level focus. Attacks target MQTT protocol behavior, not broker or device specifics. This enables use across different MQTT implementations and deployments without reliance on vendor details.
• ML-ready labels. Traffic is labelled as normal, Denial of Service, Man in the Middle, or Intrusion. This supports anomaly and intrusion detection tasks in IoT for training, validation, and testing.
• Reusable tabular format. Curated tables with a consistent schema allow direct integration into common preprocessing, feature selection, and model training pipelines.
• Benchmarking and comparability. A fixed feature set and standardized labels let researchers build baselines, compare algorithms under identical conditions, and report reproducible results.
• Broad applicability. Suitable for streaming or batch detection studies, robustness checks against protocol-level threats, feature engineering for MQTT traffic on IoT security.

## 2 Background

In [1], we studied machine learning methods to detect MQTT attacks using this dataset. Based on that use, we prepared this data article to assign a stable name (MQTT_UAD) [2] and to document how the data were built, processed, and structured for reuse.

The context is IoT security and network-based Intrusion Detection Systems. MQTT is widely used in home and industrial IoT [3,4], which makes protocol-level datasets useful for IDS training and evaluation [5].

In IoT security, several datasets support different study needs. IoT-23 [6] provides labeled benign and malicious traffic across multiple protocols and scenarios, useful for cross-protocol baselines. For MQTT-focused work, MQTT-IoT-IDS2020 [7] offers MQTT traffic from a simulated setup with attacks such as scanning and brute force. MQTT-Set [8], built with the IoT-flock framework [9], supplies MQTT traces including failed authentication, flooding, and message hold experiments. Bot-IoT [10] contributes large-scale botnet activity with DoS and DDoS and includes an MQTT weather-station case. Complementing these resources, MQTT_UAD provides protocol-level MQTT attacks captured in a real testbed together with normal traffic, intended for anomaly and intrusion detection research.

Methodologically, we set up a real MQTT environment, captured normal operation and controlled attacks, and converted PCAP to labelled CSV with a fixed field schema for common ML workflows. The data are three CSV files, one per scenario.

The attack design focuses on recognised MQTT protocol threats [11]: Denial of Service, Man-in-the-Middle, and Intrusion. The goal is to represent protocol behaviour under these threats without tying the data to a specific broker or device.

## 3 Data Description

The MQTT_UAD is composed of three files in CSV format, each corresponding to a specific type of attack. The detailed characteristics of these files are:

• **DoS.csv:** Contains 94,625 frames, of which 45,513 correspond to attack traffic and 49,112 to normal traffic.
• **MitM.csv:** Contains 110,668 frames, with 3855 frames affected by the MitM attack and 106,813 frames of normal traffic.
• **Intrusion.csv:** Contains 80,893 frames in total, of which 1898 are intrusion attacks and 78,995 are normal traffic frames.

All the files of the dataset have the same 67 fields, which are taken from the ``Wireshark Display Filter Reference'' [12] are grouped as follows:

• **Common fields**: These fields are common to all frames, independently of the use of the MQTT protocol. As non-MQTT traffic may be generated during the attacks, the inclusion of non-MQTT packages in the dataset allows the IDSs models to identify general patterns for networks that include other devices apart from the IoT devices. These fields were selected based on the study of the AWID [13] dataset, which documents attacks on IEEE 802.11 networks. A detailed description of these fields is presented in Table 1

**Table 1:** Description of common fields.

Field Name	Description	Type
frame.time_delta	Delta time from the previously captured frame	Time offset
frame.time_delta_displayed	Delta time from the previously displayed frame	Time offset
frame.time_invalid	Label a time as invalid	Label
frame.time_relative	Time elapsed since the first frame	Time offset
ip.src	Source IP address	IPv4 Address
ip.dst	Destination IP address	IPv4 Address
tcp.srcport	Source port	Unsigned integer, 2 bytes
tcp.dstport	Destination port	Unsigned integer, 2 bytes
eth.src	Source MAC address	Ethernet or other MAC address
eth.dst	Destination MAC address	Ethernet or other MAC address
frame.cap_len	Length of the captured frame	Unsigned integer, 4 bytes
frame.coloring_rule.name	Coloring rule for the name	String
frame.coloring_rule.string	Coloring rule for the string	String
frame.comment	Frame comments	String
frame.comment.expert	Expert comments	Label
frame.encap_type	Encapsulation type	Signed integer, 2 bytes
frame.file_off	File offset	Signed integer, 8 bytes
frame.ignored	If the frame is ignored	Boolean
frame.incomplete	If the frame is incomplete	Label
frame.interface_id	Interface identifier	Unsigned integer, 4 bytes
frame.interface_name	Interface name	String
frame.len	Wired frame length	Unsigned integer, 4 bytes
frame.link_nr	Link number	Unsigned integer, 2 bytes
• **MQTT protocol fields:** Fields referring to the MQTT protocol, therefore only contanining information relevant to frames using this protocol. A detailed description of these fields is presented in Table 2

**Table 2:** Description of MQTT fields.

Field Name	Description	Type
mqtt.clientid	Client ID	Character string
mqtt.clientid_len	Client ID Length	Unsigned integer, 2 bytes
mqtt.conack.flags	Acknowledge Flags	Unsigned integer, 1 byte
mqtt.conack.flags.reserved	Reserved	Boolean
mqtt.conack.flags.sp	Session Present	Boolean
mqtt.conack.val	Return Code	Unsigned integer, 1 byte
mqtt.conflag.cleansess	Clean Session Flag	Boolean
mqtt.conflag.passwd	Password Flag	Boolean
mqtt.conflag.qos	QoS Level	Unsigned integer, 1 byte
mqtt.conflag.reserved	(Reserved)	Boolean
mqtt.conflag.retain	Will Retain	Boolean
mqtt.conflag.uname	User Name Flag	Boolean
mqtt.conflag.willflag	Will Flag	Boolean
mqtt.conflags	Connect Flags	Unsigned integer, 1 byte
mqtt.dupflag	DUP Flag	Boolean
mqtt.hdrflags	Header Flags	Unsigned integer, 1 byte
mqtt.kalive	Keep Alive	Unsigned integer, 2 bytes
mqtt.len	Msg Len	Unsigned integer, 8 bytes
mqtt.msg	Message	Character string
mqtt.msgid	Message Identifier	Unsigned integer, 2 bytes
mqtt.msgtype	Message Type	Unsigned integer, 1 byte
mqtt.passwd	Password	Character string
mqtt.passwd_len	Password Length	Unsigned integer, 2 bytes
mqtt.proto_len	Protocol Name Length	Unsigned integer, 2 bytes
mqtt.protoname	Protocol Name	Character string
mqtt.qos	QoS Level	Unsigned integer, 1 byte
mqtt.retain	Retain	Boolean
mqtt.sub.qos	Requested QoS	Unsigned integer, 1 byte
mqtt.suback.qos	Granted QoS	Unsigned integer, 1 byte
mqtt.topic	Topic	Character string
mqtt.topic_len	Topic Length	Unsigned integer, 2 bytes
mqtt.username	User Name	Character string
mqtt.username_len	User Name Length	Unsigned integer, 2 bytes
mqtt.ver	Version	Unsigned integer, 1 byte
mqtt.willmsg	Will Message	Character string
mqtt.willmsg_len	Will Message Length	Unsigned integer, 2 bytes
mqtt.willtopic	Will Topic	Character string
mqtt.willtopic_len	Will Topic Length	Unsigned integer, 2 bytes
• **Frame labelling:** A special field called 'type' is used to label frames according to the type of attack they are exposed to. The labels are: 'DoS' for frames under denial-of-service attacks, 'MitM' for Man-in-the-Middle attacks, 'intrusion' for intrusions from unknown MQTT clients and 'normal' for frames not affected by any attack. This field is presented in Table 3

**Table 3:** Tagging of frames based on their state.

Field name	Description	Type
Type	Tag frames as normal or under attack	``normal'' / ``DoS'' / ``MitM'' / ``intrusion''

## 4 Experimental Design, Materials and Methods

### 4.1 MQTT environment

We set up a real testbed that combines IoT devices using the MQTT protocol with everyday user machines (laptops and smartphones). Besides exchanging MQTT messages, these machines also browse the web and use common Internet services. This mixing of IoT and non-IoT activity produces traffic that is closer to what appears in real deployments.

All traffic passes through a single router running OpenWRT, where it is captured for analysis. In the next subsections we describe the main components of the setup: the access network, the MQTT broker with its web app and API, the IoT devices (sensor and actuator), and the user machines that interact with the system.

• **The network:** A TP-link TL-WR1043ND Router is used as the access point for all the devices in the environment. The router is flashed using the OpenWRT firmware [14] and can capture all traffic generated in the network using the TCPdump tool.
• **MQTT Broker:** The Broker is the main component of the MQTT network. It manages all the massages from the nodes of the MQTT network and is responsible for the proper operation of the IoT environment. The MQTT broker is designed to support many requests, providing scalability of the system. In this work, the MQTT Broker server is developed in JavaScript using the Aedes library [15]. Additionally, on the node.js server, the ``express'' library is used to develop an API REST that facilitates communication between the application and the devices. Also, the server hosts a web application that allows the interacion between the operator and the IoT System.
• **IoT devices:** Two types of devices have been deployed in the test environment, namely a sensor and an actuator. The devices are based on the NodeMCU board, a low-cost microcontroller with an ESP8266 Wi-Fi chip and a GPIO header. This type of boards are popular in many IoT prototypes due to its compact size and ease of use. The IoT devices use the PubSubClient [16], *C*++ library to manage the communication with the MQTT network. The sensor device is equipped with an HC-SR04 ultrasonic sensor and broadcasts the sensor readings using the “distance/ultrasonic” topic. The actuator device is equipped with a relay to control a light bulb according to the Boolean messages received through the “light/relay” topic, i.e. 0 = Off, 1 = On.
• **The application:** A web application has been developed to facilitate interaction with the IoT devices. The application uses the mqtt.js library to communicate with the MQTT Broker, which makes possible to visualize the flow of messages inside the MQTT network as well as to publish new messages in a topic. Each time a connection is made, the user interface works as a client of the IoT system, which subscribes to the two topics. The application runs in a local server and can be accessed from any device connected to the Wi-Fi network and therefore can be used to visualize the sensor device readings and to change the actuator device state.
• **End-user devices:** Non IoT related devices such as computers or smartphones may be present in the test environment. These devices can be used to interact with the Web application, and therefore with the IoT environment.

### 4.2 Attacks performed in the test environment

The environment presented in the previous section has been used to perform a series of attacks on the IoT devices in the network. During the attacks, all traffic passing through the router was captured for further analysis. A detailed description of the attacks performed is presented in the following sections.

### 4.3 Denial of service attack (DoS)

In an IoT environment using the MQTT protocol, the Broker server is responsible for the management of all connections among the clients. Therefore, the Broker is one of the main targets in a DoS attack because if the Broker becomes unavailable and cannot resolve legitimate requests, the whole IoT environment will stop working [17].

**Fig. 1:**
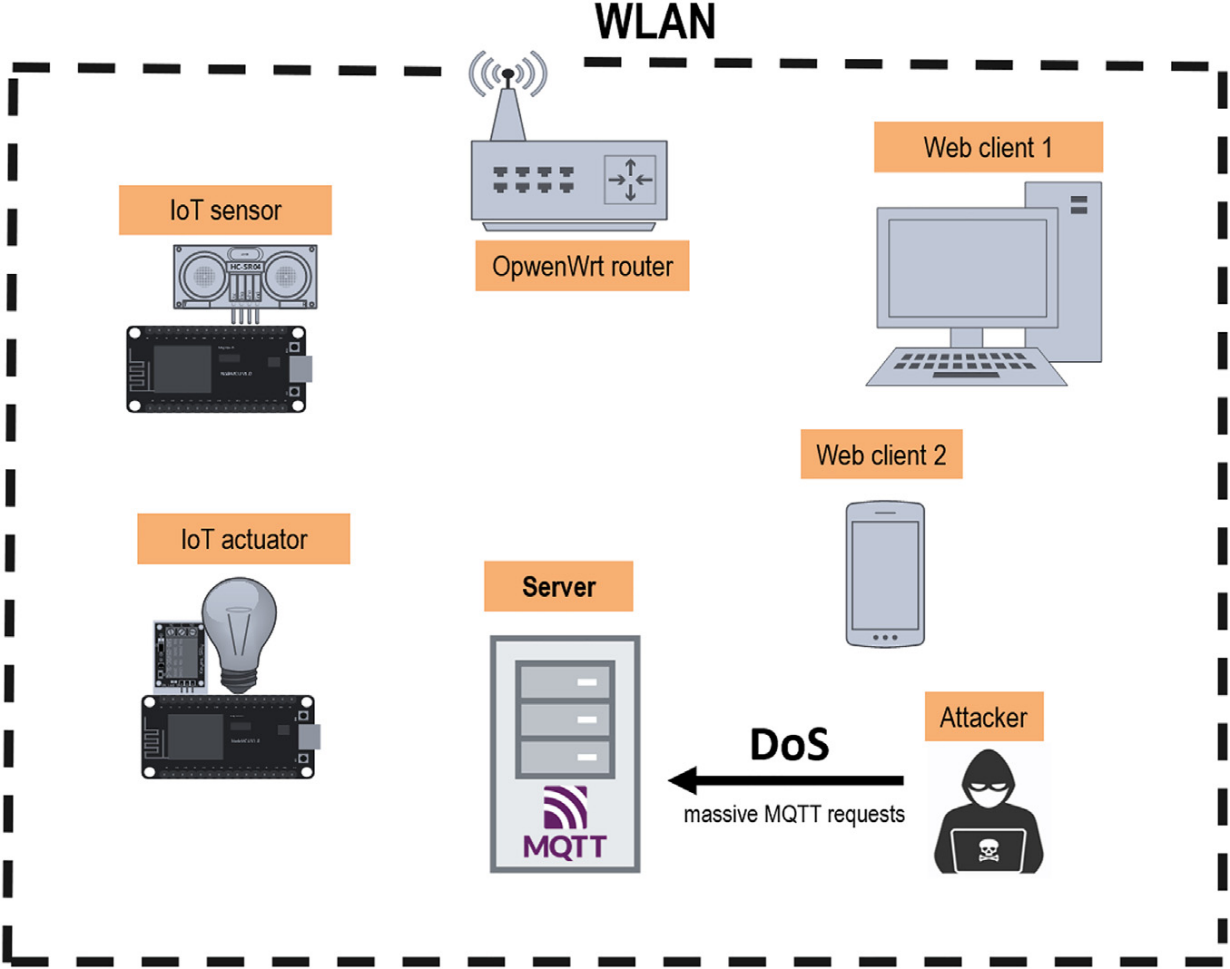
MQTT DoS attack arquitecture.

An attacker can exploit these vulnerabilities and exhaust both clients and the Broker of a network. In this work, we have performed an attack on the IoT environment using the “mqtt-Malaria” [18] tool, which allows to the simulation of several MQTT clients which can publish defined size messages at a defined rate. We simulated 1000 clients which publish 1000 messages every 100 milliseconds against the broker.The result is the exhaustion of the Broker resources, who is no longer able to process the legitimate requests by the devices on the network. Fig. 1 presents a diagra of the DoS attack performed.

### 4.4 Man-in-the-middle attack

Man in the Middle (MitM) is a type of attack in which a malicious agent anonymously analyses the traffic on a network. The attacker places between two devices that expect to communicate directly with each other and forwards the messages while analysing them. In this work, the attacker first scans the network and detects the use of the MQTT protocol for the well-known port 1886. The attacker then places between the sensor device and the Broker by using the “ ettercap}” tool to perform an ARP spoofing attack which will cause the Broker and the IoT sensor to communicate with the attacker.

**Fig. 2:**
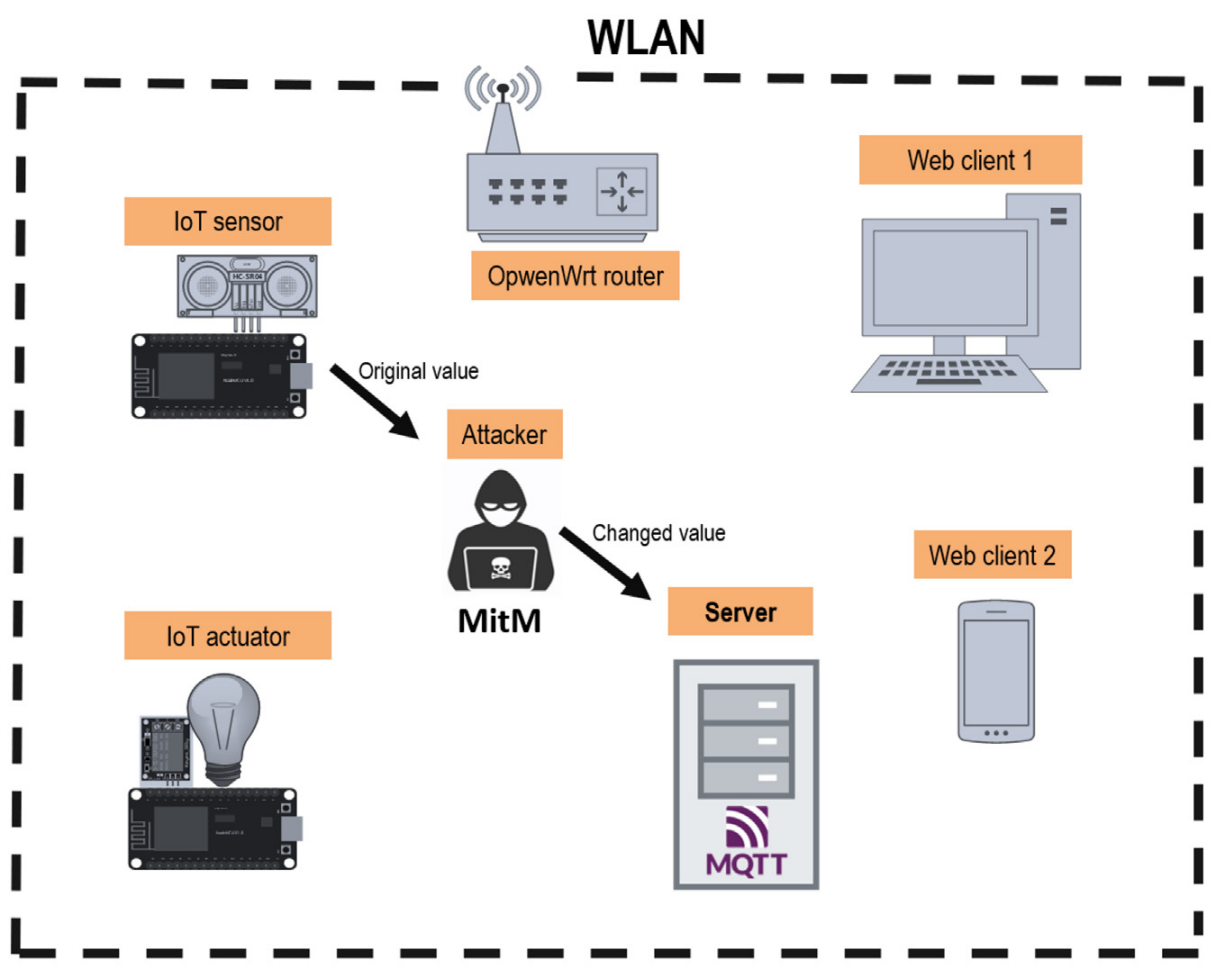
MQTT MitM attack structure.

Once placed between the devices in the network, the attacker filters the MQTT traffic on the network using the nfqsed tool [19] to modify the reading value of the sensor device. The modified frame is then forwarded to the Broker, which will accept the message without realizing it has been modified by the attacker. Fig. 2 presents a diagram of the MitM attack performed.

### 4.5 Intrusion attack

An unauthorized MQTT client connects to the broker, subscribes to a broad topic such as # to receive messages across topics as permitted by MQTT topic syntax, and publishes messages to inject traffic. This targets protocol-level configuration and access control rather than broker-specific features. Publicly reachable MQTT services on the well-known port (for example, TCP 1883) can be found via Internet-wide device search engines like Shodan [20], which enables discovery and unsolicited connections.

Once an attacker detects and connects to a non-secure MQTT network, the topic '\#' can be use as the topic name to which the attacker is subscribed. This connection will return any message published in any topic of the network. Therefore, the attacker would have successfully performed an intrusion attack on the environment.

**Fig. 3:**
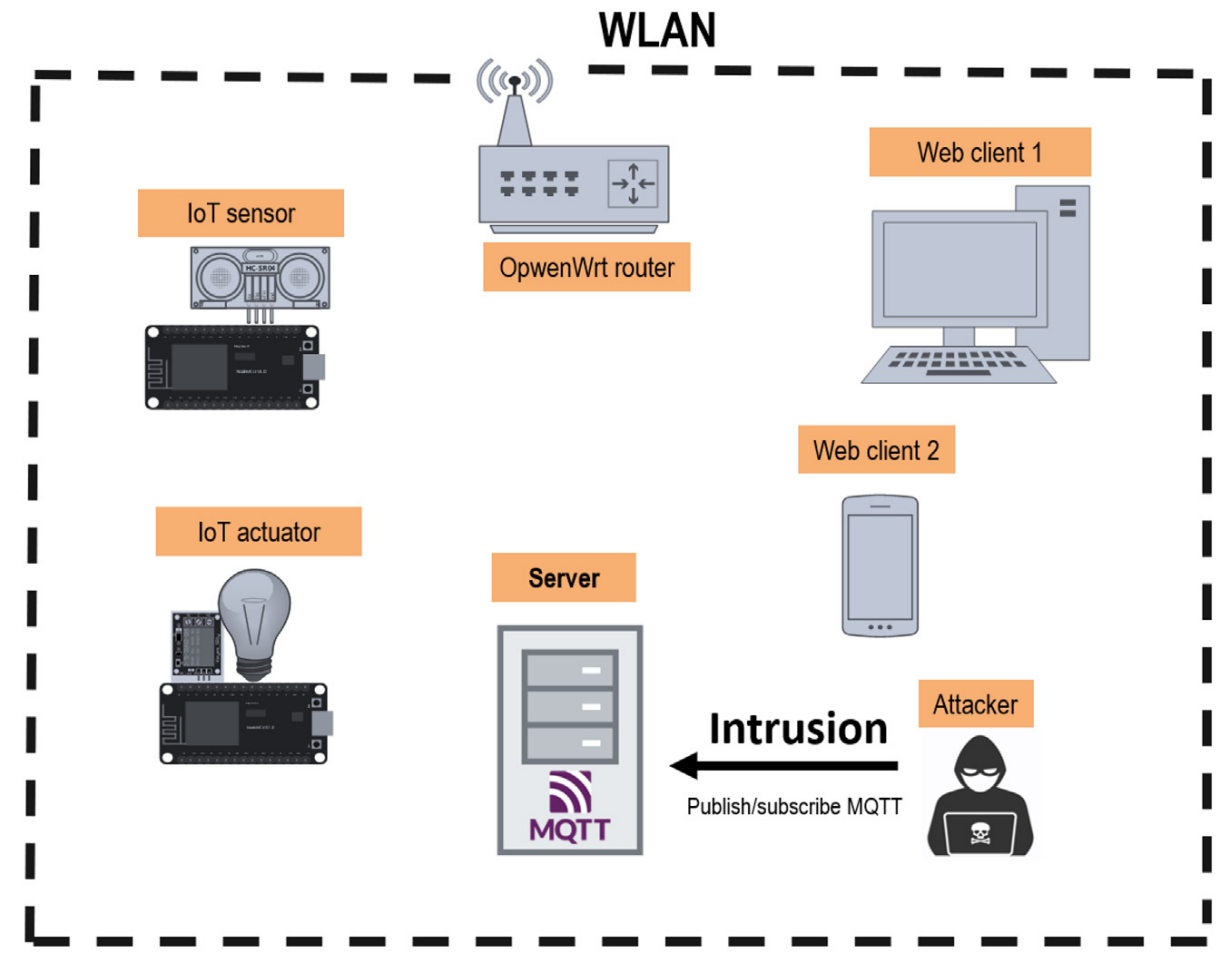
MQTT Intrusion attack.

In this work, the “mosquito” MQTT client is used to perform the intrusion attack on the IoT network. Once the attacker has analysed the traffic for the “distance/ultrasonic” and “light/relay” topics, fake messages are inserted on the network for both topics. Fig. 3 presents a diagram of the MitM attack performed.

## Limitations

Not applicable

## Ethics Statement

The authors have read and follow the ethical requirements for publication in Data in Brief. This work does not involve human subjects, animal experiments, or data collected from social media platforms.

The dataset was generated in a controlled laboratory testbed and contains only technical network traffic and MQTT protocol fields. No personally identifiable information (PII) is included.

## Credit Author Statement

**Jose Aveleira-Mata:** created the dataset, verified the labels, and wrote the manuscript. **Martín Bayón-Gutiérrez:** validation, Writing- Reviewing and Editing. **María Teresa García-Ordás:** Data curation, Visualization, Validation, Writing- Reviewing and Editing. **Isaías García-Rodríguez:** Conceptualization, Supervision, Writing- Reviewing and Editing. **Natalia Prieto-Fernández:** supervision, Writing- Reviewing and Editing. **Héctor Alaiz-Moretón:** provided overall guidance during conceptualization and dataset creation and reviewed the manuscript.

## Data Availability

FigshareMQTT_UAD: MQTT Under Attack Dataset. A public dataset for the detection of attacks in IoT networks using MQTT protocol (Original data).
